# RNA Is an Integral Component of Chromatin that Contributes to Its Structural Organization

**DOI:** 10.1371/journal.pone.0001182

**Published:** 2007-11-14

**Authors:** Antonio Rodríguez-Campos, Fernando Azorín

**Affiliations:** Institute of Molecular Biology of Barcelona (IBMB), CSIC, and Institute for Research in Biomedicine (IRB), Parc Científic de Barcelona, Barcelona, Spain; Centre National de la Recherche Scientifique, France

## Abstract

Chromatin structure is influenced by multiples factors, such as pH, temperature, nature and concentration of counterions, post-translational modifications of histones and binding of structural non-histone proteins. RNA is also known to contribute to the regulation of chromatin structure as chromatin-induced gene silencing was shown to depend on the RNAi machinery in *S. pombe*, plants and *Drosophila*. Moreover, both in *Drosophila* and mammals, dosage compensation requires the contribution of specific non-coding RNAs. However, whether RNA itself plays a direct structural role in chromatin is not known. Here, we report results that indicate a general structural role for RNA in eukaryotic chromatin. RNA is found associated to purified chromatin prepared from chicken liver, or cultured *Drosophila* S2 cells, and treatment with RNase A alters the structural properties of chromatin. Our results indicate that chromatin-associated RNAs, which account for 2%–5% of total chromatin-associated nucleic acids, are polyA^−^ and show a size similar to that of the DNA contained in the corresponding chromatin fragments. Chromatin-associated RNA(s) are not likely to correspond to nascent transcripts as they are also found bound to chromatin when cells are treated with α-amanitin. After treatment with RNase A, chromatin fragments of molecular weight >3.000 bp of DNA showed reduced sedimentation through sucrose gradients and increased sensitivity to micrococcal nuclease digestion. This structural transition, which is observed both at euchromatic and heterochromatic regions, proceeds without loss of histone H1 or any significant change in core-histone composition and integrity.

## Introduction

In eukaryotes, histones pack DNA into chromatin, a periodic structure of regularly spaced nucleosomes [1]. In the nucleosome, 145–147 bp of DNA are wrapped around a protein core formed by two copies of each histone H2A, H2B, H3 and H4. This evolutionarily conserved nucleoprotein complex is evenly distribute, every 200±40 bp, all throughout the eukaryotic genome [2]. Much is known about nucleosome structure as it was determined at atomic resolution [3]–[5]. In addition to core histones, higher eukaryotes contain linker histone H1. Histone H1 contributes to folding of the nucleosomes into higher-order chromatin structures, which are stabilized by the interaction of both histone H1 and the core histone N-terminal tails with the linker DNA [6]–[9]. Therefore, histone-DNA interactions determine both the basic structural properties of the nucleosome as well as the formation of higher-order chromatin structures [10]. Multiple factors, however, are known to influence both nucleosome structure and higher-order chromatin structures [11], [12]. These include pH, temperature, ionic-strength, DNA bendability, histone modifications and, in particular, binding of structural non-histone proteins (i.e., HP1, Polycomb), which often depends on specific post-translational modifications of the histones N-terminal tails [13]. Several results indicate that RNA also influences chromatin structure as, first, chromatin-induced gene silencing depends on the RNAi machinery [14]–[16] and, second, dosage compensation requires the contribution of specific non-coding RNAs [17], [18]. A role of RNA in the formation of higher-order heterochromatin structures was also proposed as, in mammals, HP1 *foci* are sensitive to treatment with RNase A being recovered upon the addition of total RNA or hnRNA, but not by the addition of tRNA or bacterial mRNA [19]. Altogether, these observations indicate a contribution of RNA to the regulation of chromatin structure and function. Whether RNA itself plays a direct structural role in chromatin is, however, not known.

Here, we report results indicating that RNA plays a general structural role in eukaryotic chromatin. Our results indicate that purified chromatin contains significant amounts of RNA (2%–5% of total nucleic acids). Whether this chromatin-associated RNA(s) contributes to chromatin structure was addressed by analyzing the sedimentation behavior through linear sucrose gradients of native oligonucleosomal fragments, purified from chicken liver or cultured *Drosophila* S2 cells, before and after treatment with RNase A. Density gradient centrifugation is a powerful method to separate and analyze macromolecules [20]. A classical application of sedimentation in sucrose gradients is the analysis of repetitive nucleoprotein structures such as polysomes [21], [22] and nucleosomes [23]. Soluble chromatin fragments, obtained by mild micrococcal nuclease digestion of purified nuclei, are resolved by density gradient centrifugation into fractions of homogeneous composition and precise molecular entity, from mononucleosomes to oligonucleosomes [20]. Our results show that, upon treatment with RNase A, chromatin fragments display a clear shift towards the lighter zone of the gradient, which is abolished in the presence of the specific RNase inhibitor, antiRNase. Decreased sedimentation is observed for bulk chromatin as well as for chromatin at specific genomic locations, either euchromatic or heterochromatic, and is accompanied by an increased sensitivity to MNase digestion. Altogether, these results indicate that RNA is an integral component of chromatin that contributes to its structural organization.

## Results

### Purified chromatin contains RNA

**Figure 1:**
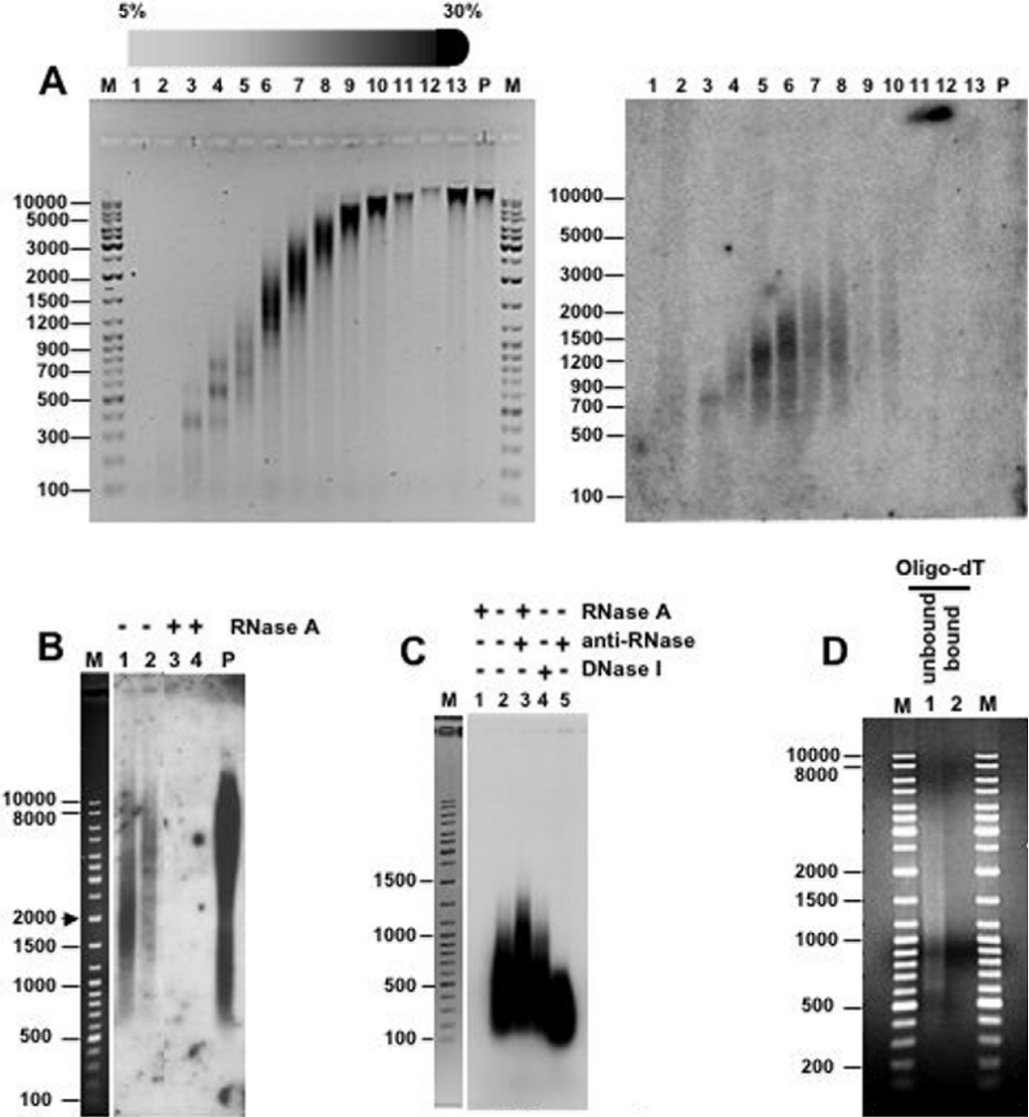
Purified chicken liver chromatin contains RNA. A) Chicken liver chromatin was prepared by micrococcal nuclease digestion of purified nuclei and then subjected to sedimentation through a linear 5%–30% sucrose gradients. After centrifugation, 1ml fractions were collected, subjected to total nucleic acids extraction and analyzed by electrophoresis in 1% agarose-TBE gels (left panel). In parallel, chromatin fractions were subjected to RNA extraction using Ultraspec™ RNA Isolation System (Biotecx) and analyzed by Northern blotting in a glyoxal-1% agarose gel using high molecular weight genomic chicken DNA as a probe (right panel). Fraction numbers are indicated. Lanes M correspond to molecular weight markers. B) Chromatin fractions 7 and 10 of the gradient shown in A) were subjected to RNA extraction as indicated above, treated with RNase A (lanes 3 and 4) or not (lanes 1 and 2), and analyzed by Northern blotting as in A). Lane M, corresponds to molecular weight markers. Lane P, corresponds to the probe used. C) Chromatin from a mixture of fractions 7, 8 and 9 of the gradient shown in A) were subjected to RNA extraction and either untreated (lane 2), treated with RNase A in the absence (lane 1) or in the presence of anti-RNase (Ambion) (lane 3), or treated with DNase I (Roche) (lane 4). After phenol extraction and isopropanol precipitation, samples were ^32^P-labeled by reverse transcription with Omniscript® RT Kit (Qiagen) (2 h at 37°C) using a mixture of hexanucleotides of random sequence. Samples were then analyzed in a 1% agarose-TBE gel, blotted and the membrane directly exposed. Lane M, corresponds to molecular weight markers. D) Chromatin-associated RNA was purified and incubated with oligo-dT immobilized resin (Oligotex™ mRNA Purification System, QIAGEN). After elution, bound (lane 2) and unbound material (lane1) were analyzed in a glyoxal-1% agarose, 10 mM sodium phosphate (pH 6,8) gel. Lanes M correspond to molecular weight markers.

As shown in Figure 1, purified chicken liver chromatin contains RNA. In these experiments, chicken liver chromatin was prepared by micrococcal nuclease (MNase, Sigma) digestion of isolated nuclei that were purified by centrifugation through a sucrose cushion to avoid as much as possible contamination with cytoplasmic RNA. After MNase digestion, soluble chromatin was fractionated by centrifugation through a linear 5%–30% sucrose gradient and chromatin fractions were subjected to total nucleic acids extraction or to selective RNA extraction using Ultraspec™ RNA Isolation System (Biotecx). Density gradient centrifugation results in the typical distribution of chromatin fragments in which more dense fractions contain longer oligonucleosomal fragments (Figure 1A, left). Selective RNA extraction from these purified chromatin fractions renders significant amounts of product as determined by Northern blotting using total high-weight chicken genomic DNA as a probe (Figure 1A, right). As shown in Figure 1, the Ultraspec-extracted material shows an average size very similar to that of the DNA contained in the same chromatin fraction. The Ultraspec-extracted material is sensitive to digestion with RNase A. In this experiment, fractions 7 and 10 of the gradient shown in Figure 1A were subjected to selective RNA extraction and, then, treated with RNase A, or not, prior to Northern analysis (Figure 1B). After RNase A treatment, no hybridization signal is observed. To further confirm its RNA nature, the material obtained after RNA extraction from a mixture of fractions 7, 8 and 9 of the same gradient (Figure 1A, left) was radioactively labeled by reverse transcription in the absence of any treatment (Figure 1C, lane 2) or after treatment with RNase A (Figure 1C, lane 1). No labeling can be observed upon RNase A treatment. On the contrary, strong labeling is observed when RNase A digestion was carried out in the presence of the RNase inhibitor anti-RNase (Ambion) (Figure 1C, lane 3) or when the material was subjected to digestion with DNase I (Figure 1C, lane 4). Altogether, these results indicate that purified chicken liver chromatin contains significant amounts of RNA, which accounts for around 2% to 5% of total nucleic acids content (see Materials and Methods for the quantitative determination of the RNA content of purified chromatin). As shown in Figure 1D, the vast majority of chromatin-associated RNA(s) is not bound by oligo-dT affinity resin, indicating that it is polyA^−^.

**Figure 2:**
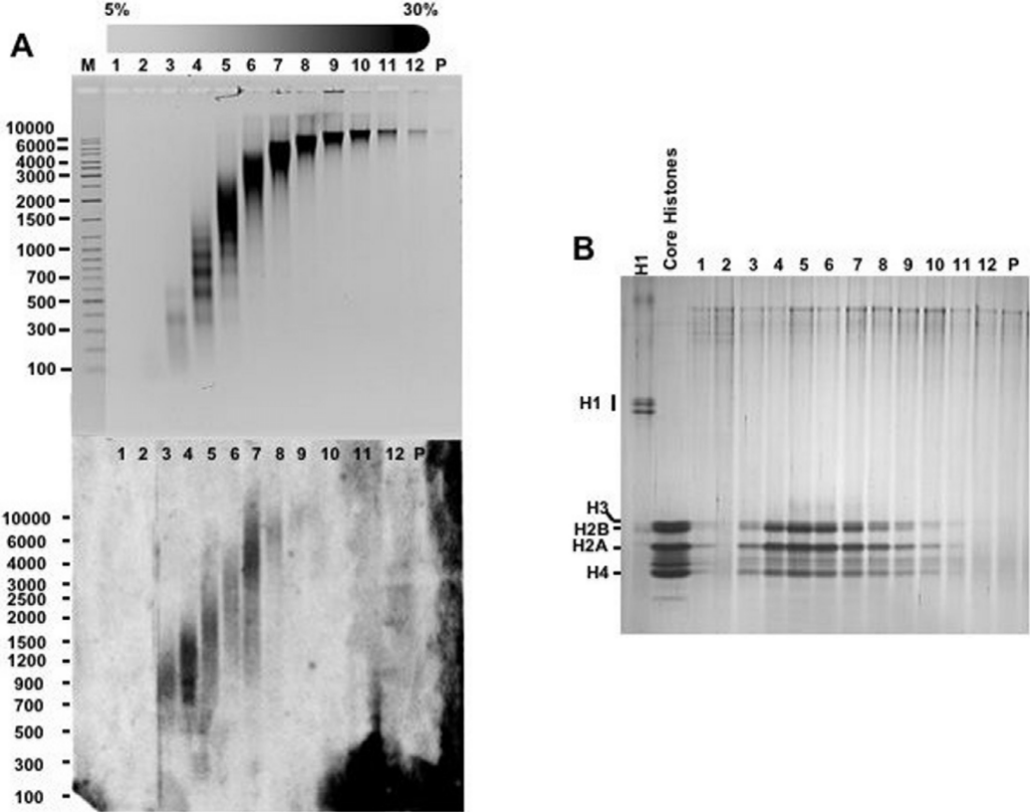
RNA remains associated to chromatin at high ionic strength. A) Chicken liver chromatin was prepared by micrococcal nuclease digestion of purified nuclei and then subjected to sedimentation through a linear 5%–30% sucrose gradients containing 0,65 M NaCl. After centrifugation, fractions were collected and their DNA (top) and RNA (bottom) content determined as in Figure 1. Fraction numbers are indicated. Lane M, corresponds to molecular weight markers. B) The histone content of each fraction was analyzed by SDS-PAGE (lanes 1-P). As controls, H1 from calf thymus and hydroxylapatite-purified core histones from chicken liver are also presented. The gel was stained with silver.

In the experiments described above, sucrose gradient centrifugation was performed at low ionic strength (0,2 mM EDTA). RNA, however, remains associated to chromatin at high ionic strength (Figure 2). In these experiments, chicken liver chromatin was fractionated by centrifugation through a linear 5%–30% sucrose gradient containing 0,65 M NaCl. At this ionic strength, histone H1 is known to be released from chromatin [11], [20] and, accordingly, no histone H1 was detected when the protein content of chromatin fractions prepared at 0,65 M NaCl was analised by SDS-PAGE electrophoresis (Figure 2B). Under these conditions, however, chromatin fractions contained significant amounts of RNA (Figure 2A), indicating that RNA associates to chromatin tighter than histone H1.

### Treatment with RNase A alters the sedimentation behavior of chromatin

**Figure 3:**
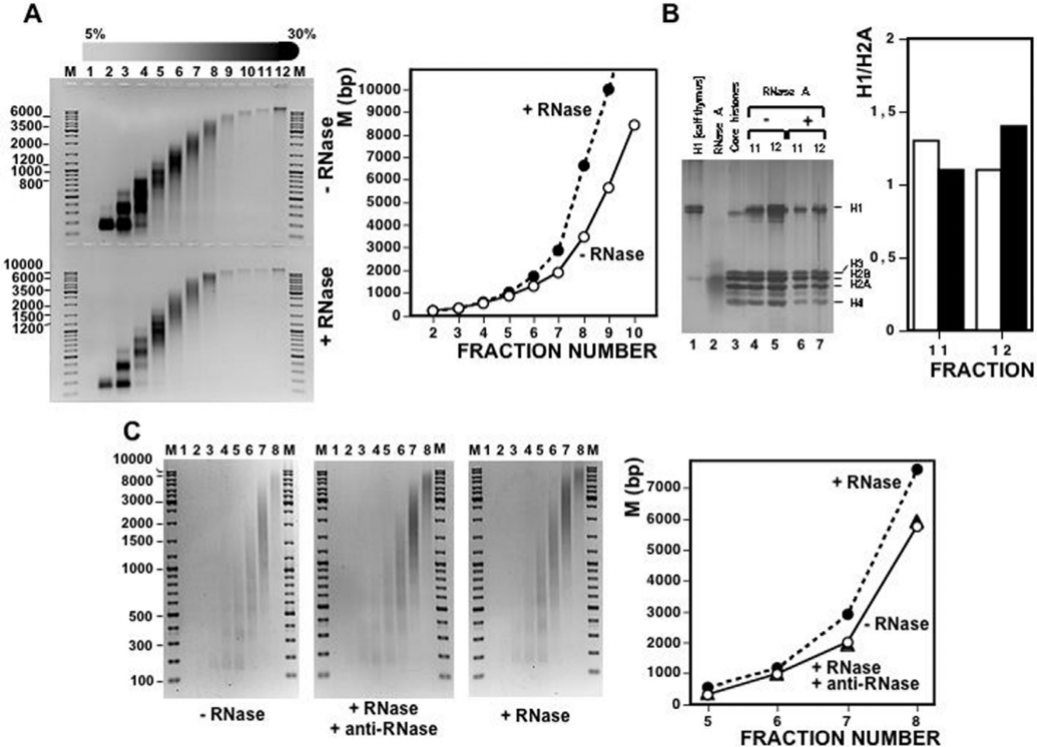
Treatment with RNase A alters the sedimentation behavior of purified chicken liver chromatin. A) Chicken liver chromatin, prepared by micrococcal nuclease digestion of purified nuclei, was subjected to sedimentation through a linear 5%–30% sucrose gradient after treatment with RNase A (bottom panel) or not (top panel). After centrifugation and fractionation, samples were subjected to total nucleic acids extraction and analyzed by electrophoresis in 1% agarose-TBE gels. Fraction numbers are indicated. Lanes M correspond to molecular weight markers. Quantitation of the results is shown on the right where the average molecular weight (M) of the chromatin fragments contained in each fraction, expressed as bp of DNA, is presented as a function of the fraction number: (°) untreated chromatin, (•) chromatin treated with RNase A. B) Histone content of chromatin fractions 11 and 12 of the gradients shown in A) was analyzed by SDS-PAGE: untreated chromatin (lanes 4 and 5), chromatin treated with RNase A (lanes 6 and 7) and, as controls, H1 from calf thymus (lane 1), RNase A (lane 2) and hydroxylapatite-purified core histones from chicken liver (lane 3). The gel was stained with silver. Quantitation of the results is shown on the right where the H1/H2A ratio of fractions 11 and 12 is presented before (white columns) and after (black columns) treatment with RNase A. C) The sedimentation behavior of chicken liver chromatin was determined before (left panel) and after treatment RNase A either in the presence of anti-RNase (Ambion) (central panel) or in the absence of any added inhibitor (right panel). Fraction numbers are indicated. Lanes M, correspond to molecular weight markers. Quantitation of the results is shown on the right: (°) untreated chromatin, (▴) chromatin treated with RNase A in the presence of anti-RNase, (•) chromatin treated with RNase A in the absence of any added inhibitor.

In these experiments, prior to sedimentation through linear 5%–30% sucrose gradients, bulk chicken liver chromatin obtained by mild MNase digestion of purified nuclei was either digested with RNase A (Figure 3A, bottom panel) or not (Figure 3A, top panel). As shown in Figure 3A, equivalent fractions of the same density contain chromatin fragments of higher molecular weight when chromatin is digested with RNase A than when it is not. This effect increases with the size of the nucleosomal fragments (Figure 3A, graph), so that chromatin fragments of up to 1.000 bp of DNA show no significant change in sedimentation after treatment with RNase A. On the contrary, chromatin fragments of molecular weight >3.000 bp of DNA show a strong change in sedimentation after digestion with RNase A. Decreased sedimentation observed after treatment with RNase A requires actual degradation of an RNA component as it is not observed when treatment with RNase A is carried out in the presence of the specific RNase A inhibitor anti-RNase (Figure 3C). Moreover, the shift in the sedimentation rate observed upon treatment with RNase A is not a consequence of the loss or degradation of the histone component (Figure 3B). In these experiments, chromatin from fractions 11 and 12 of the gradients shown in Figure 3A was precipitated by the addition of MgCl_2 _and NaCl, and the pellets were dissolved, loaded and electrophoresed in a PAGE-SDS gel. No significant change in histone composition or integrity is detected after treatment with RNase A (Figure 3B, compare lanes 4 and 5 with lanes 6 and 7). In particular, treatment with RNase A does not result in any significant change in histone H1 content (Figure 3B, graph). This observation is especially relevant as it is known that the degree of compactness of chromatin and, therefore, its sedimentation rate is strongly dependent on the presence of histone H1 [24], [25].

### Treatment with RNase A alters chromatin structure at both euchromatic and heterochromatic regions

**Figure 4:**
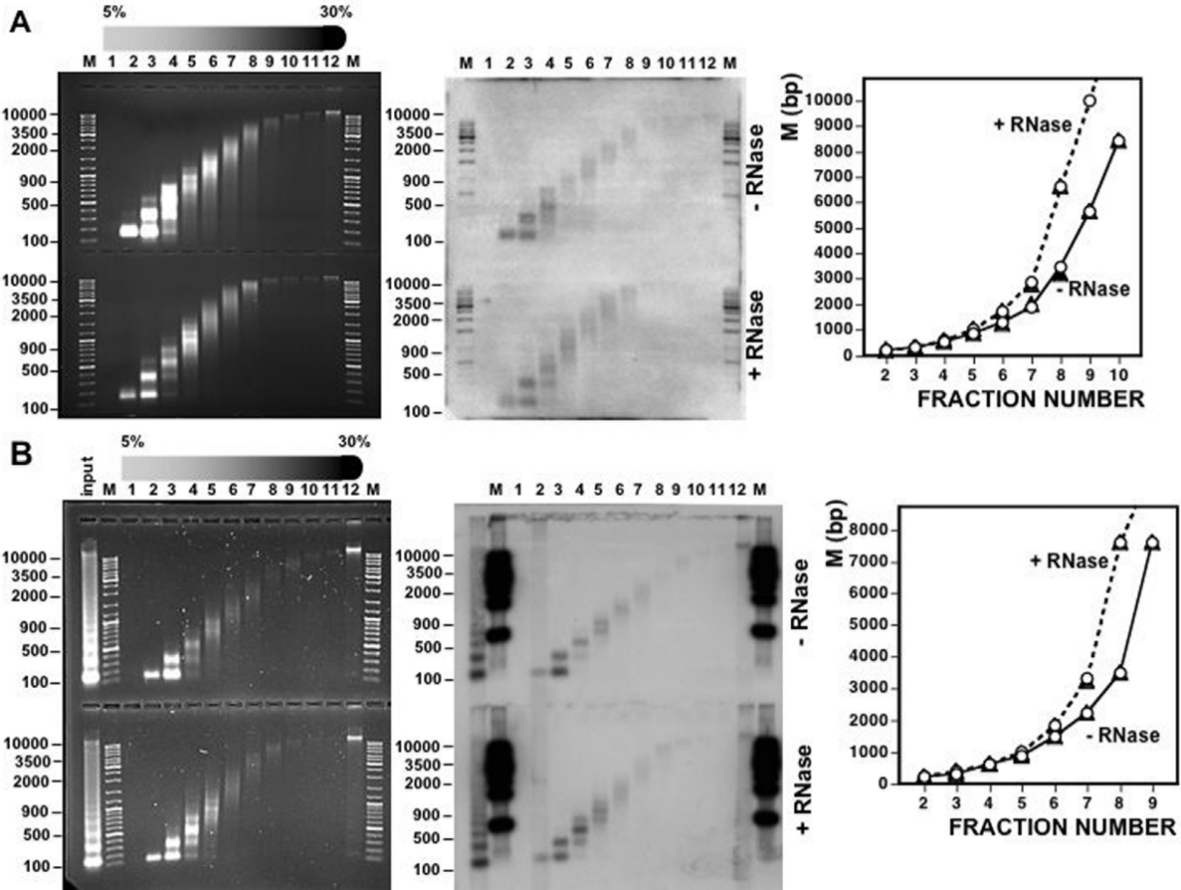
Treatment with RNase A alters the sedimentation behavior of chicken liver chromatin at specific genomic regions. The effect of treatment with RNase A on the sedimentation behavior of chromatin was determined for bulk chicken liver chromatin (left panels) and for chromatin at specific genomic locations (right panels) by Northern analysis of the gels on the left using specific probes for the *Pax3 locus* A) and the chicken 41–42 bp centromeric satellite B). Fraction numbers are indicated. Lanes M, correspond to molecular weight markers. Quantitation of the results is shown on the right of each panel: (°) bulk chromatin, (▴) chromatin of the *Pax3 locus* A) or the chicken 41–42 bp centromeric satellite B).

Results shown above indicate that treatment with RNase A reduces the sedimentation rate of bulk chicken liver chromatin. Similar results were obtained when the effects of treatment with RNase A on the chromatin structure of specific genomic regions were determined by Southern blotting using specific probes (Figure 4). In these experiments, two specific genomic regions were analyzed, the euchromatic *Pax3 locus* [26] (Figure 4A) and the heterochromatic 41–42 bp centromeric chicken satellite [27] (Figure 4B). In both cases decreased sedimentation is observed upon treatment with RNase A. In fact, changes in sedimentation observed at these specific genomic locations are undistinguishable from those observed with bulk chromatin (Figure 4A and 4B, graphs).

**Figure 5:**
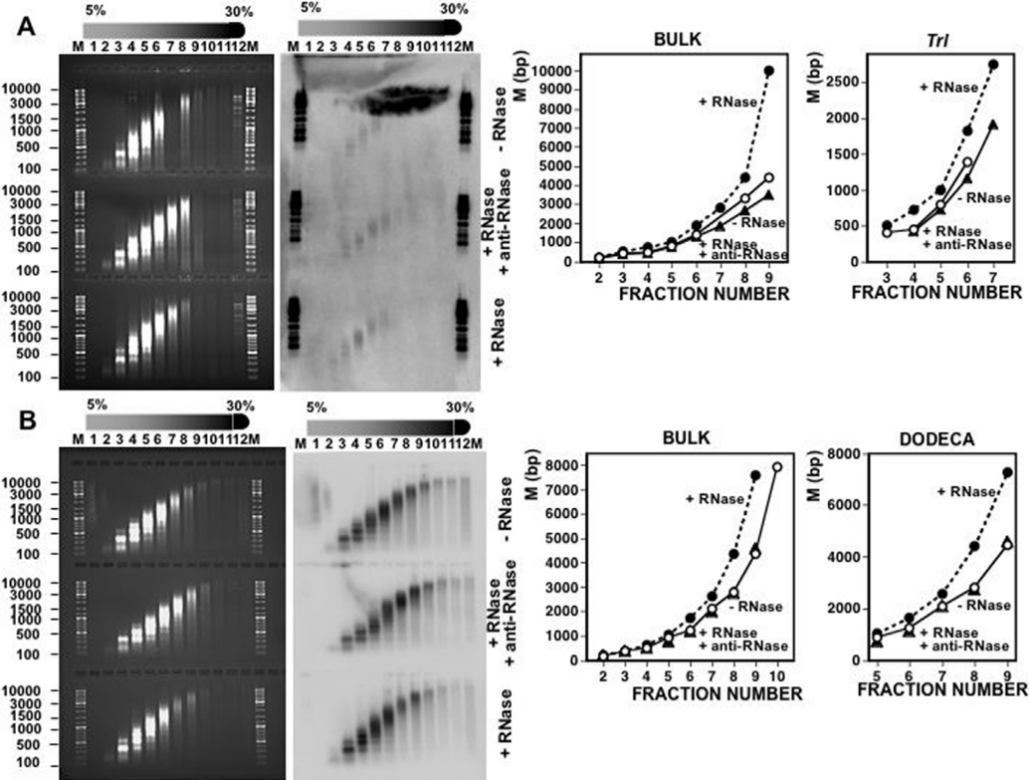
Treatment with RNase A alters the sedimentation behavior of purified *Drosophila* S2 chromatin. The effect of treatment with RNase A on the sedimentation behavior of chromatin prepared from *Drosophila* S2 cells was determined for bulk chromatin (left panels) and for chromatin at specific genomic locations (right panels) by Southern analysis of the gels on the left using specific probes for the *Trl locus* A) and the centromeric *Drosophila* dodeca-satellite B). Fraction numbers are indicated. Lanes M, correspond to molecular weight markers. Quantitation of the results is shown on the right of each panel for bulk chromatin and chromatin of the *Trl locus* A) or the centromeric dodeca-satellite B): untreated chromatin (°); chromatin treated with RNase A in the presence of anti-RNase (Ambion) (▴) or in the absence of any added inhibitor (•).

**Figure 6:**
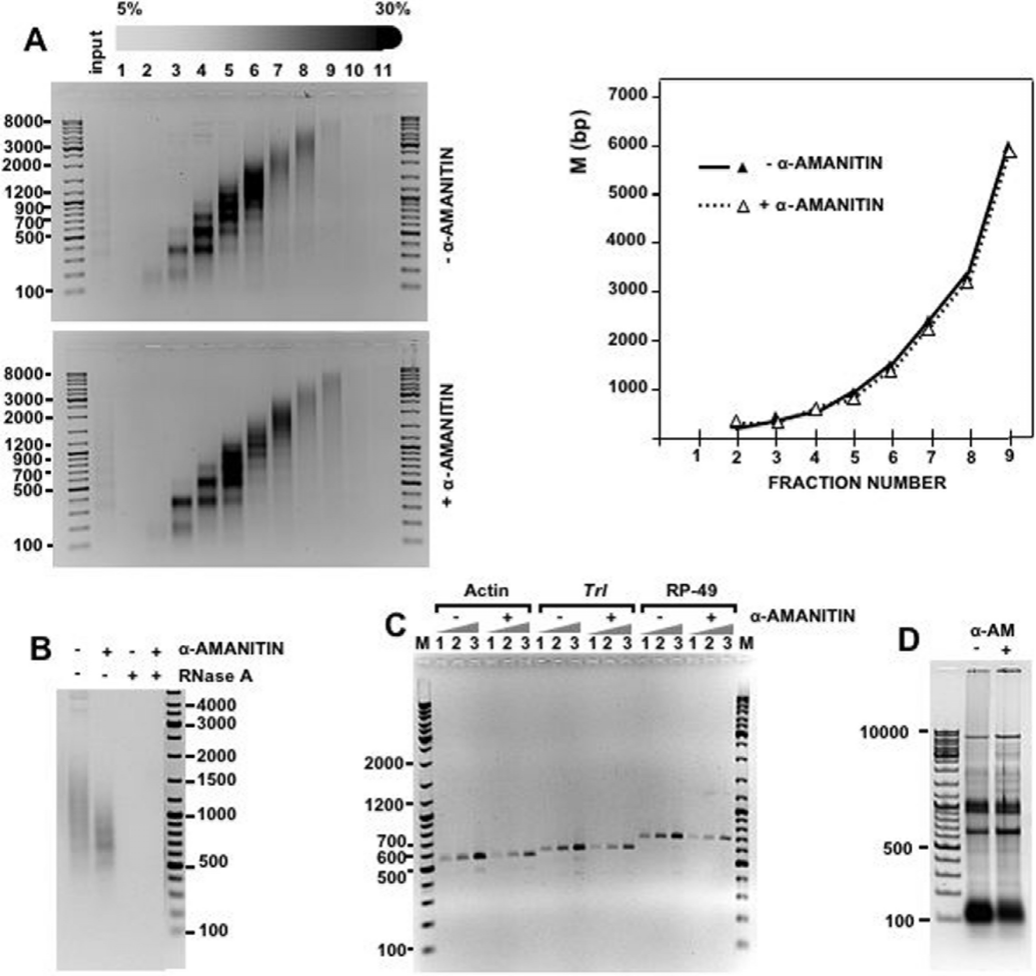
Treatment with α-amanitin does not alter the sedimentation behavior of purified *Drosophila* S2 chromatin. Prior to nuclei isolation and chromatin purification, S2 cells were either treated for 36 h with 0,2 µg/ml α-amanitin or not. A) S2 chromatin from untreated (top) and treated cells (bottom) was purified by micrococcal nuclease digestion of purified nuclei and subjected to sedimentation through linear 5%–30% sucrose gradients as described above. Quantitation of the results is shown on the right where the average molecular weight (M) of the chromatin fragments contained in each fraction, expressed as bp of DNA, is presented as a function of the fraction number for chromatin prepared from untreated (▴) and treated cells (Δ). B) Chromatin fractions 4, 5 and 6 of the gradients shown in A) obtained from cells treated with α-amanitin (lanes 2 and 4) or not (lanes 1 and 3), were subjected to RNA extraction and analised in a glyoxal-1% agarose-sodium phosphate gel before (lanes 1 and 2) and after treatment with RNase A (lanes 3 and 4). Lane M corresponds to molecular weight markers. C and D) Analysis of the efficiency of treatment with α-amanitin. D) Total RNA was prepared from treated (lane 1) or untreated cells (lanes 2), and 5 µg of each were analysed in a 1% agarose-TBE native gel. Lane M corresponds to molecular weight markers. C) Determination of the levels of nascent transcripts encoding GAGA (*Trl*), actin 5C and RP-49. Increasing amounts of total RNA (0,1, 0,2 and 0,5 µg, lanes 1-3), prepared from untreated (lanes -) and treated cells (lanes +), were analysed by RT-PCR (Omniscript® RT Kit, QIAGEN) as indicated under Materials and Methods using appropriate primers to specifically amplify fragments of the Actin 5C (585 bp), *Trl* (662 bp) and RP-49 (702 bp) genes. Amplified fragments were analysed in a 1% agarose-TBE gel. Lanes M correspond to molecular weight markers.

Similar results were obtained when chromatin was prepared from cultured *Drosophila* S2 cells (Figure 5). Also in this case, purified chromatin contains RNA (Figure 6B) and treatment with RNase A decreases sedimentation of bulk S2-chromatin as well as of chromatin of two specific genomic locations, the euchromatic *Trl locus* (Figure 5A) and the heterochromatic centromeric dodeca-satellite (Figure 5B). As with chicken liver chromatin, no decreased sedimentation is observed when treatment with RNase A is carried out in the presence of anti-RNase (Figure 5).

### Treatment with α-amanitin does not affect association of RNA to chromatin

To address the question of whether chromatin-associated RNA(s) corresponds to nascent RNA transcripts, we analysed the effects of blocking transcription by treatment with α-amanitin [28] (Figure 6). In these experiments, *Drosophila* S2 cells were treated for 36 h with 0,2 µg/ml of α-amanitin as described under Materials and Methods. To determine the effectiveness of this treatment, the levels of nascent RNAs encoding GAGA (*Trl*), Actin 5C and RP-49 were determined by RT-PCR (Figure 6C). In all three cases, treatment with α-amanitin results in an approximately 3 to 4-fold reduction on the levels of the corresponding nascent RNAs. After treatment, chromatin was digested with MNase and analysed by sedimentation through 5%–30% linear sucrose gradients. As shown in Figure 6A, treatment with α-amanitin does not significantly affect the sedimentation rate of purified chromatin. Moreover, after treatment with α-amanitin, the RNA content of purified chromatin is similar to that observed in chromatin prepared from untreated cells (Figure 6B). These results indicate that chromatin-associated RNA(s) are not likely to correspond to nascent transcripts.

### RNA-depleted chromatin displays a higher sensitivity to MNase digestion

**Figure 7:**
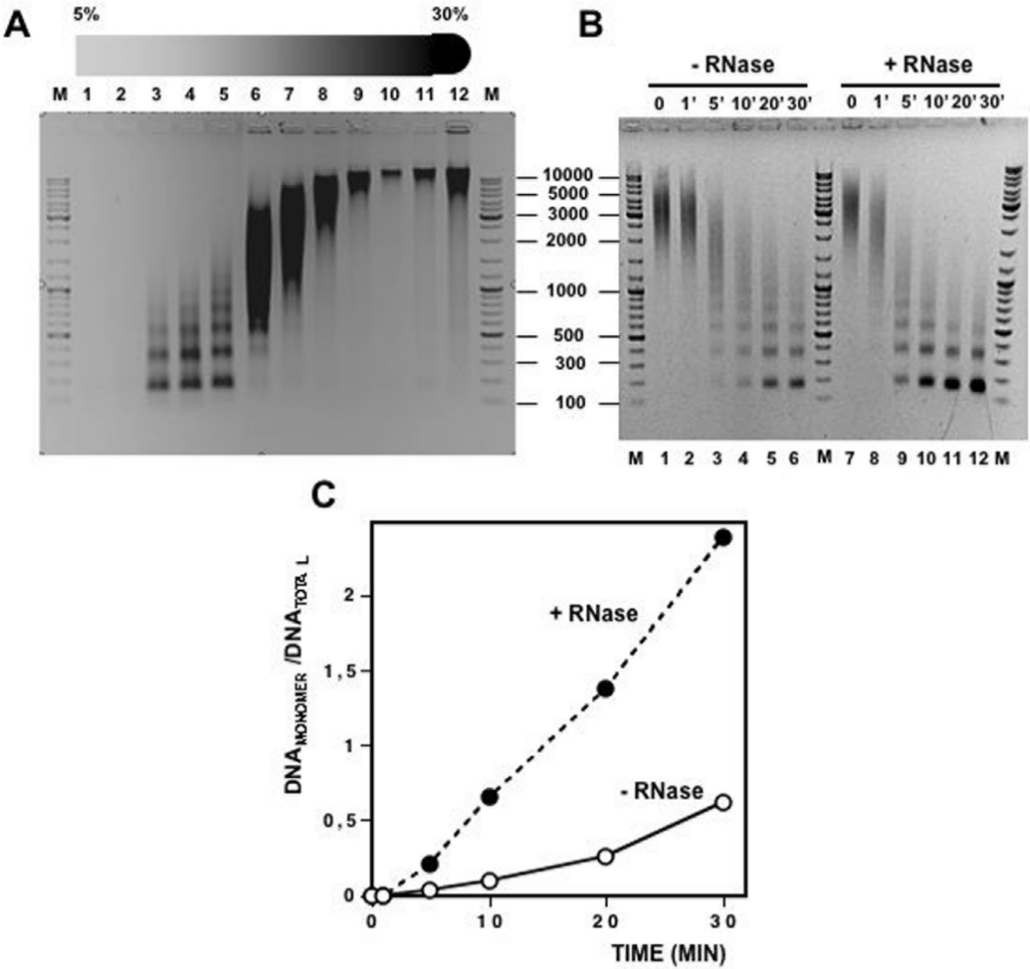
Treatment with RNase A increases the sensitivity of chicken liver chromatin to cleavage by micrococcal nuclease. A) Preparative 5%–30% linear sucrose gradient of chicken liver chromatin. Fraction numbers are indicated. Lanes M, correspond to molecular weight markers. B) Fraction 8 of the gradient shown in A) was treated with RNase A (lanes 7–12) or not (lanes 1–6) and, then, digested at 37°C with 0.4 units of micrococcal nuclease (Sigma) at increasing times as indicated. After micrococcal nuclease digestion, samples were deproteinized and analyzed by electrophoresis in 1% agarose-TBE gels. Lanes M correspond to molecular weight markers. C) Quantitation of the results shown in B). The ratio of mononucleosomal DNA versus total DNA is presented as a function of the digestion time for untreated chromatin (°) and chromatin treated with RNase A (•).

**Figure 8:**
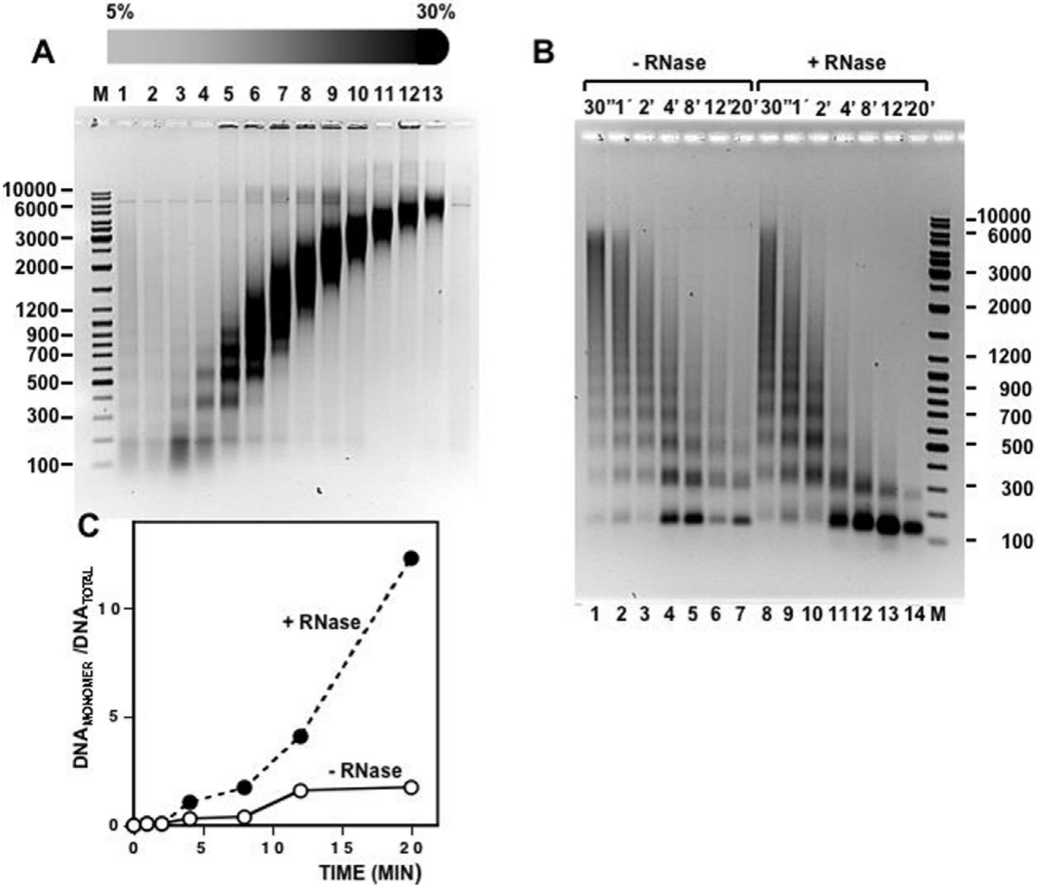
Treatment with RNase A increases the sensitivity of purified *Drosophila* S2 chromatin to cleavage by micrococcal nuclease. A) Preparative 5%–30% linear sucrose gradient of chromatin from *Drosophila* S2 cells. Fraction numbers are indicated. Lane M corresponds to molecular weight markers. B) Fraction 13 of the gradient shown in A) was treated RNase A (lanes 8 to 14) or not (lanes 1–7), and, then, digested at 37°C with 0,4 units of micrococcal nuclease (Sigma) at increasing times as indicated. After micrococcal nuclease digestion, samples were deproteinized and analyzed by electrophoresis in 1% agarose-TBE gels. Lane M corresponds to molecular weight markers. C) Quantitation of the results shown in B). The ratio of mononucleosomal DNA versus total DNA is presented as a function of the digestion time for untreated chromatin (°) and chromatin treated with RNase A (•).

Decreased sedimentation observed after treatment with RNase A is likely the consequence of a reduction on the degree of compactness of chromatin. Consistent with this hypothesis, accessibility to digestion by MNase increases after RNA depletion (Figure 7). In these experiments, chromatin prepared by mild MNase digestion of isolated nuclei was purified by centrifugation through a linear 5%–30% sucrose gradient (Figure 7A) and chromatin from fraction 8 that, in average, contains oligomers of about 25 nucleosomes (∼5.000 bp of DNA) was either treated with RNase A or not, and, then, subjected to further MNase digestion for increasing times (Figure 7B). Upon treatment with RNase A, sensitivity to cleavage by MNase strongly increases as reflected by the increased production of mononucleosomal fragments obtained after equivalent times of digestion (Figure 7C). Similar results were obtained when chromatin was prepared from cultured *Drosophila* S2 cells (Figure 8). Also in this case, treatment of S2-chromatin with RNase A increases its accessibility to digestion with MNase.

## Discussion

Here, we report evidence supporting the physical association of RNA to chromatin purified by sucrose gradient centrifugation. Sucrose gradient centrifugation is used for the purification of discrete molecular entities from complex mixtures of macromolecules. Purification is achieved on the basis of the differential sedimentation rates of the various components, which depend on their mass, density and shape [20]. In particular, sucrose gradient centrifugation is extensively used for the purification of large multimeric complexes such as chromatin. On the appropriate conditions of pH and ionic strength, chromatin contained in a particular fraction is free of soluble components other than those directly associated to the native nucleoprotein complex. In our experiments, chromatin was obtained by mild MNase digestion of isolated nuclei that, to avoid as much as possible contamination with cytoplasmic RNA, were purified by centrifugation through a sucrose cushion. Our results show that chromatin purified by sucrose gradient centrifugation contains significant amounts of RNA. This conclusion arises from the fact that, when purified chromatin is subjected to selective RNA extraction, a nucleic acid residue is obtained, which hybridizes to radioactively labeled total high-weight genomic DNA (Figure 1A) and is sensitive to digestion with RNase A (DNase and protease-free) (Figures 1B and 1C). These chromatin-associated RNAs, which account for 2%–5% of the total nucleic acids content of chromatin, are likely to correspond to long single-stranded molecules as, on one hand, they are digested by RNase A and, second, their size is similar to that of the DNA isolated from the same chromatin fractions (Figures 1A and 2A). At this respect, it must be noticed that MNase cleaves both DNA and single-stranded RNA [29]. Therefore, the actual size the chromatin-associated RNAs is likely to be higher than that observed after MNase digestion. What is the nature of these chromatin-associated RNAs? Our results indicate that they are not likely to correspond to nascent transcripts as they are also observed when cells are treated with α-amanitin (Figure 6B). The fact that RNA remains associated to chromatin at high ionic strength (Figure 2), and that silent heterochromatic regions appear to contain RNA(s), also argue against this possibility. Our data also show that the vast majority of these chromatin-associated RNAs are polyA^−^ (Figure 1D), indicating that they do not correspond either to mature RNA-polII transcripts. It is possible that these chromatin-associated RNAs would correspond to a class of non-coding RNAs. Increasing evidence indicates that a majority of the eukaryotic genome, including non-coding regions, is transcribed to RNA. It was recently shown that over 85% of the *Drosophila melanogaster* genome is transcribed [30]. In the recent years, non-coding RNAs have focused considerable interest due to their regulatory properties. Most of the regulatory non-coding RNAs identified to date are relatively small (micro-RNAs, siRNAs) [31], [32] but a few long non-coding RNAs have also been identified. These include *Xist* (18 kb long), which is responsible for silencing of the X-chromosome [33], and *Air* (>100 kb long), which silences the paternal *Igf2r/Slc22a2/Slc22a3* gene [34]. Most likely, long non-coding RNAs are more frequent than previously anticipated. Actually, a recent analysis in mouse revealed the existence of multiple nuclear long non-coding RNAs (>10 kb) of unknown function [35]. Whether these long non-coding RNAs associate to chromatin remains to be determined but it is possible that some of them would play a structural role in chromatin as such described here.

Treatment with RNase A alters the sedimentation behavior of chromatin through sucrose gradients (Figure 3A). In density gradients, particles sediment according to their sedimentation coefficient (*s*). As *s* depends on molecular weight, density and shape, the analysis of the sedimentation behavior could yield ambiguous information. But, when two of these parameters are constant, the change in the sedimentation rate actually reflects the variation in one particular property; for particles of similar mass and density, the value of *s* depends only on their shape [20]. Centrifugation through sucrose gradients has been extensively used to analyze structural changes in chromatin [36], [37]. Actually, identification of the nucleosome as the repeating unit of chromatin was made possible in part from sedimentation analysis of chromatin prepared by mild MNase digestion of purified nuclei [23], [24]. Further analysis demonstrates that the selective removal of the linker histone H1 produces a significant decrease in sedimentation rate due to unfolding of the chromatin fiber [24], [25]. Our results indicate that treatment of purified chromatin with RNase A decreases its sedimentation rate through sucrose gradients. The magnitude of the change in sedimentation observed is much higher than that expected from the reduction of mass associated to the degradation of the RNA component of chromatin. RNA accounts only for 2%–5% of the total nucleic acids content of purified chromatin. Therefore, full degradation of chromatin associated RNAs would result in 1%–2,5% loss of total chromatin mass, a reduction too small to be detected by sucrose gradient centrifugation. Therefore, as with H1-depleted chromatin, decreased sedimentation likely reflects that treatment with RNase A reduces the degree of compactness of chromatin. Actually, the change in sedimentation observed for RNA-depleted chromatin is similar to that of H1-depleted chromatin (compare Figures 2A and 3A). Consistent with this hypothesis, accessibility to MNase cleavage increases after treatment with RNase A (Figures 7 and 8). This structural transition is not due to a change in histone composition or integrity (Figure 3B). In particular, after treatment with RNase A, histone H1 remains bound to the chromatin fiber. Moreover, decreased sedimentation requires actual degradation of an RNA component as it is abolished in the presence of anti-RNase, a specific RNase inhibitor. Altogether, these results indicate that, similar to depletion of histone H1, degradation of chromatin-associated RNA(s) changes the global compactness of the chromatin fiber. This structural transition is observed both for bulk chromatin as well as for chromatin at specific genomic locations, either euchromatic or heterochromatic, suggesting that the association of RNA is a general characteristic of chromatin. What is the molecular basis of the association of RNA to chromatin and its contribution to chromatin structure? Our results suggest that RNA is tightly associated to chromatin as it is resistant to treatment at high ionic strength that, on the other hand, releases linker histone H1. Moreover, preliminary results indicate that, upon total nucleic acids extraction, RNA is found bound to DNA (not shown). These results suggest that the association of RNA to chromatin occurs *via* DNA. On the other hand, several indications suggest that RNA might stabilize binding of structural non-histone proteins to chromatin. For instance, binding of HP1 to heterochromatin is sensitive to digestion with RNase A [19] and it requires the contribution of the hinge domain, which is known to bind RNA *in vitro* [38]. It is possible that RNA also influences binding of other structural non-histone proteins. Binding of RNA to chromatin might also stabilizes high-order chromatin structures by facilitating folding of the chromatin fiber, or long-distance chromatin-chromatin interactions, through its simultaneous binding to multiple sites. Further work is neccesary to understand the precise structural role that RNA plays in chromatin.

## Materials and Methods

### Isolation of nuclei

All plasticware, glassware and solutions were sterilized by autoclaving. Fresh chicken livers were purchased on the public food market. *Drosophila* S2 cells were grew by conventional methods. Nuclei were isolated by Dounce homogenization of minced material (when liver) or spun cells (when S2) in 0,5 M sucrose, 0,5% Triton X-100, 100 µg/ml Phenyl Methane Sulfonyl Fluoride (PMSF), TES buffer (15 mM Tris-HCl pH 7,4, 60 mM KCl, 0,15 mM spermine, 0,5 mM spermidine, 2 mM EDTA, 1 mM DTT). The homogenate was diluted with three volumes of 2,3 M sucrose in TES and layered over a 5 ml cushion of 2,3 M sucrose in TES. After centrifugation in a Beckman SW28 rotor (27.000 rpm, 4°C, 3 h), pelleted nuclei were washed twice with cold TES, precipitated in a Beckman JA-25.50 rotor (5.000 rpm, 10 min, 4°C) and resuspended in the same buffer. The DNA concentration was measured at 260 nm and the nuclei, usually at 0,5–1 mg/ml, were frozen at −80°C after the addition of Dimethyl sulfoxide (DMSO) to a 10% final concentration.

### Preparation of native chromatin

Native chromatin was obtained by mild digestion with micrococcal nuclease (MNase) of isolated nuclei in 3 mM CaCl_2 _during different times and the reaction stopped by addition of EDTA (50 mM final concentration). After pelleting in an Eppendorf tabletop centrifuge (8.000 rpm, 2 min, 4°C), the nuclei were disrupted by pipetting in 0,2 mM EDTA and spun again; the soluble chromatin was recovered from the supernatant and DNA concentration was measured at 260 nm. On average, up to 30–40% of the starting material was recovered as soluble chromatin.

### RNase A treatment

Treatment with RNaseA (DNase and protease-free) (Roche) was performed using 0,5 to 1 µg of enzyme. When indicated, RNaseA activity was inhibited by the addition of 100 to 200 units of anti-RNase (Ambion) following manufacturer instructions.

### RNA isolation and quantitation

RNA was extracted using Ultraspec™ RNA Isolation System (Biotecx) according to manufacturer instructions. Glyoxal-agarose gels were performed on a NaOH-treated electrophoresis cell. To minimize RNA degradation, agarose solution, electrophoresis and loading buffers were autoclaved. When the amount of RNA associated to purified chromatin was determined, chromatin purified by sucrose gradient sedimentation was deproteinized overnight at 37°C with 0,2% SDS, 0,5 mg/ml proteinase K, followed by phenol extraction and ethanol precipitation. The precipitated sample was resuspended in sterile water and total nucleic acid content determined at 260 nm using a quartz cuvette extensively treated with 2N NaOH and sterile water. The sample was, then, divided into three aliquots, containing 6 µg of total nucleic acid, which were treated at 37°C for 2 h with 20 µg of DNAse I (RNase-free), with 20 µg of RNase A (DNase and protease-free) or not treated. After these treatments, nucleic acid content of serial 10-fold dilutions of each sample was determined with RiboGreen® RNA Quantitation Reagent (Molecular Probes) in a fluorescence microplate reader (excitation at 480 nm, emission at 520 nm) in comparison to both DNA and RNA standards. In parallel, another aliquot of deproteinized chromatin was extracted with Ultraspec™ RNA Isolation System and the purified material was divided in two portions, incubated for 2 h at 37°C with 20 µg of RNase A (DNase and protease-free) or not, and processed as before.

### Sedimentation analysis through linear sucrose gradients

Linear sucrose gradients were performed with a linear gradient maker. Prior to their use, the mixing chambers and polyvinyl tubing were treated during 1 h with a 2N NaOH solution and then washed with sterile water. To prevent proteases activities, PMSF was added to a 100 µg/ml final concentration to the sucrose solutions. 5%–30% sucrose (dissolved in 5 mM HEPES-NaOH pH 7,4, 0,2 mM EDTA) preparative gradients containing 1 to 5 mg of soluble native chromatin were centrifuged on Beckman SW28 rotor at 22.000 rpm during 14 h at 4°C. Analytical gradients containing 100–200 µg were run in a Beckman SW41 rotor (22.000 rpm, 14 h, 4°C).

### Isolation of RNA poly A-

The polyadenylated status of purified chromatin-associated RNA(s) was analysed by affinity chromatography using an oligo-dT resin (Oligotex™ mRNA Purification System, QIAGEN). Bound (poly A+) and unbound (poly A-) samples were purified and electrophoresed on glyoxal-agarose denaturant gel.

### Treatment with α-amanitin

*Drosophila* S2 cells were grew until confluence and divided in two equal portions of 300 ml of suspended cells; one of them was incubated with α-amanitin (Sigma) (0,2 µg/ml of final concentration) during 36 h at 24°C. An aliquot (5 ml) of each cellular culture was processed for total RNA isolation and their nucleic acid content determined at 260 nm using a quartz cuvette extensively treated with 2N NaOH and sterile water. Native chromatin isolation and sedimentation analysis of samples incubated or not with α-amanitin was performed as described before. Efficiency of transcriptional inhibition by α-amanitin was measured by retrotranscription using Omniscript® RT Kit (QIAGEN). RT-reactions were performed with different amounts of RNA templates using a mixture of hexanucleotides as primers. After RT, samples were phenolized, precipitated with ethanol at room temperature and, then, subjected to PCR with *Taq* polymerase (Biotools) using specific primers derived from intron and adjacent exon sequences for actin 5C, *Trl* and RP-49 genes. Primers used were: for actin 5C, 5′-TATCACTACCGTTTGAGTTC-3′, corresponding to the first exon (positions +2 to +22) and 5′-CGTGACACGCCCACATCAGC-3′, corresponding to the first intron (positions +567 to +587); for *Trl,* 5′-TGGATCTAAGACTTCGGTCC-3′, corresponding to the second exon (positions +3208 to +3228) and 5′- AGCAACTCATTCCTTCCTTG-3′, corresponding to the second intron (positions +3850 to +3870) and, for RP-49, 5′- CATGTTATCAATGGTGCTGC-3′, corresponding to the first exon (positions +20 to +40) and 5′-GAATTATGCATTAGTGGGAC-3′, corresponding to the second intron (positions +702 to +722). The size of the expected fragments are 585 bp, 662 bp and 702 bp for actin 5C, *Trl* and RP-49, respectively.
